# METTL3 as a novel diagnosis and treatment biomarker and its association with glycolysis, cuproptosis and ceRNA in oesophageal carcinoma

**DOI:** 10.1111/jcmm.18195

**Published:** 2024-03-01

**Authors:** Xu‐Sheng Liu, Yu Zhang, Zi‐Yue Liu, Yan Gao, Ling‐Ling Yuan, Dao‐Bing Zeng, Fan Tan, Hua‐Bing Wan, Zhi‐Jun Pei

**Affiliations:** ^1^ Department of Nuclear Medicine, Hubei Provincial Clinical Research Center for precision Diagnosis and Treatment of liver cancer Taihe Hospital, Hubei University of Medicine Shiyan China; ^2^ Hubei Provincial Clinical Research Center for Umbilical Cord Blood Hematopoietic Stem Cells Taihe Hospital, Hubei University of Medicine Shiyan China; ^3^ Department of Pathology Taihe Hospital, Hubei University of Medicine Shiyan China

**Keywords:** ceRNA, cuproptosis, glycolysis, METTL3, oesophageal carcinoma

## Abstract

METTL3 has been shown to be involved in regulating a variety of biological processes. However, the relationship between METTL3 expression and glycolysis, cuproptosis‐related genes and the ceRNA network in oesophageal carcinoma (ESCA) remains unclear. ESCA expression profiles from databases were obtained, and target genes were identified using differential analysis and visualization. Immunohistochemistry (IHC) staining assessed METTL3 expression differences. Functional enrichment analysis using GO, KEGG and GSEA was conducted on the co‐expression profile of METTL3. Cell experiments were performed to assess the effect of METTL3 interference on tumour cells. Correlation and differential analyses were carried out to assess the relationship between METTL3 with glycolysis and cuproptosis. qRT‐PCR was used to validate the effects of METTL3 interference on glycolysis‐related genes. Online tools were utilized to screen and construct ceRNA networks based on the ceRNA theory. METTL3 expression was significantly higher in ESCA compared to the controls. The IHC results were consistent with the above results. Enrichment analysis revealed that METTL3 is involved in multiple pathways associated with tumour development. Significant correlations were observed between METTL3 and glycolysis‐related genes and cuproptosis‐related gene. Experiments confirmed that interfered with METTL3 significantly inhibited glucose uptake and lactate production in tumour cells, and affected the expression of glycolytic‐related genes. Finally, two potential ceRNA networks were successfully predicted and constructed. Our study establishes the association between METTL3 overexpression and ESCA progression. Additionally, we propose potential links between METTL3 and glycolysis, cuproptosis and ceRNA, presenting a novel targeted therapy strategy for ESCA.

## 1 INTRODUCTION

In recent years, as a common malignant tumour, the incidence rate and mortality of oesophageal carcinoma (ESCA) have shown a worrying global upward trend. The highly invasive and early imperceptible characteristics of ESCA pose significant challenges in its diagnosis and treatment.^1^ Therefore, finding new treatment strategies and targets is crucial for improving the management and prognosis of ESCA. The current treatment methods mainly include surgical resection, radiotherapy and chemotherapy, but the results are not ideal, especially for advanced patients.^2^, ^3^, ^4^ Therefore, it is particularly important to find new treatment strategies with high efficiency and selectivity.

As a core component of m6A methylation, Methyltransferase 3 and N6‐Adenosine‐Methyltransferase Complex Catalytic Subunit (METTL3) is involved in the regulation of various biological processes, including cell proliferation, differentiation and metabolism.^5^, ^6^, ^7^, ^8^, ^9^, ^10^, ^11^, ^12^, ^13^ By regulating the m6A modification level of the target gene, METTL3 can affect the translation efficiency and post transcriptional modification of mRNA, thereby having a significant impact on cell function and physiological processes.^14^, ^15^ Research has shown that METTL3 is overexpressed in various tumours and is closely related to the biological behaviours of tumour cell proliferation, invasion and metastasis.^16^, ^17^, ^18^, ^19^, ^20^, ^21^, ^22^ In previous studies,^23^ we found that METTL3 is overexpressed in ESCA, and its expression level is closely related to ^18^F‐FDG PET/CT metabolic parameters. These results suggest its potential role in this disease.

At the molecular level, specific signalling pathways, regulatory factors and tumour suppressor genes have been found to play important roles in ESCA.^2^, ^3^ These findings provide a strong foundation for finding new treatment strategies and targets. For example, some studies have shown key signalling pathways targeting the proliferation, invasion and metastasis of tumour cells, such as PI3K/Akt, MAPK and Wnt signalling pathway may become new therapeutic targets.^24^, ^25^, ^26^ The abnormal activation of glycolytic pathways in tumour cells has been widely reported and is closely related to key processes such as tumour growth, invasion and metastasis.^27^ On the contrary, cuproptosis is a cell death mechanism closely related to intracellular copper ion levels.^28^, ^29^ Studies have found that competing endogenous RNA (ceRNA) has important regulatory functions in biology, including regulating gene expression, affecting signalling pathways, regulating the cell cycle and apoptosis.^30^, ^31^ Previous studies have found that glycolysis, cuproptosis and ceRNA play important roles in the progression of ESCA. However, there is little in‐depth research on the role of METTL3 in ESCA, especially the relationship between METTL3 and glycolysis, cuproptosis and ceRNA in ESCA.

**FIGURE 1:**
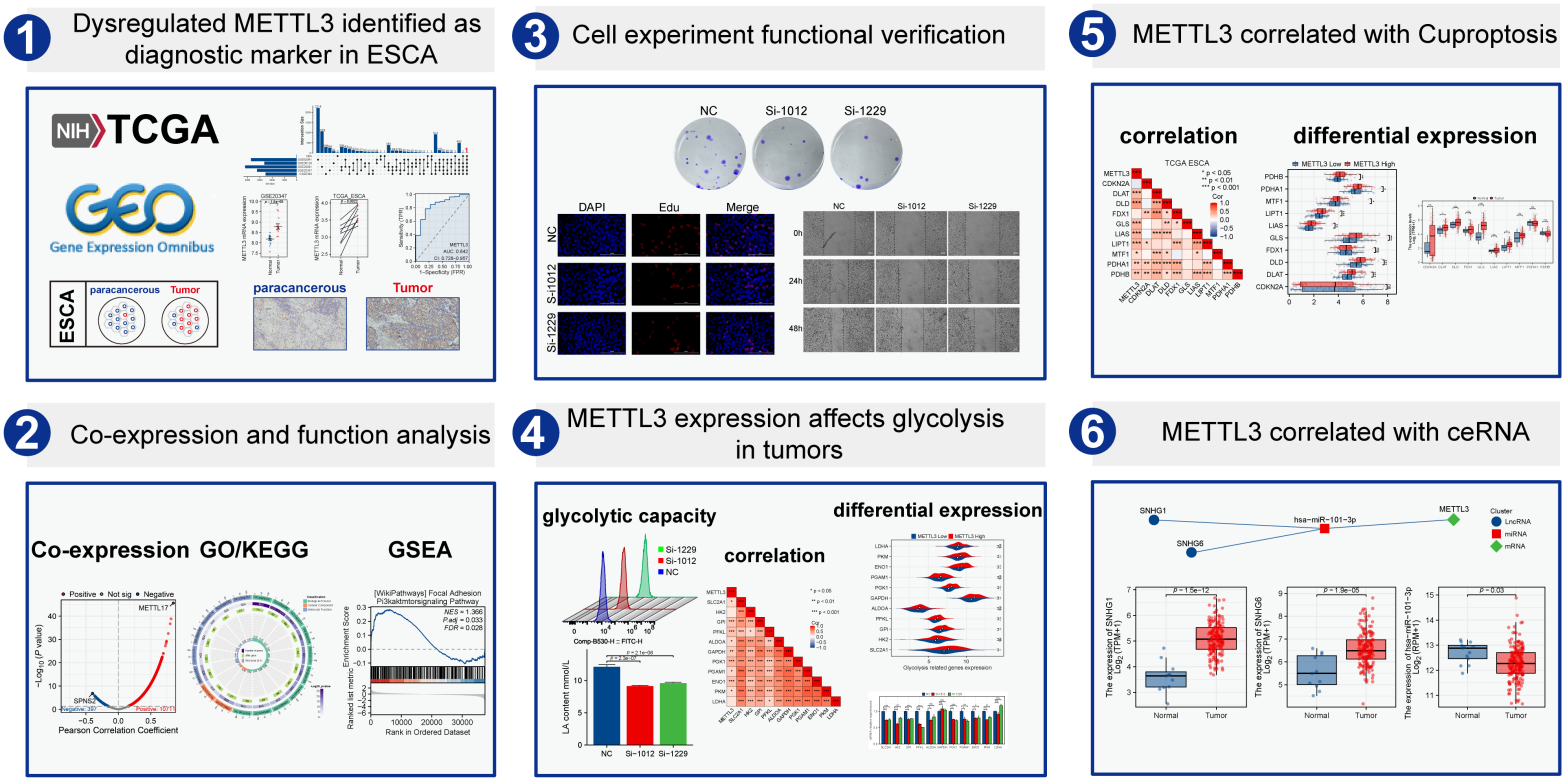
Research flow chart.

In this study, The Cancer Genome Atlas (TCGA) program and Gene Expression Omnibus (GEO) dataset queues were downloaded and processed. We analysed and used multiple databases and online websites. The differential expression of METTL3 in ESCA was confirmed through multiple dataset analysis, immunohistochemistry (IHC) staining experiments and in vitro cell experiments. We studied the co expressed gene network of METTL3 in ESCA and investigated the possible biological functions and signalling pathways involved in these genes. Finally, the potential relationship between METTL3 and tumour cell glycolysis, cuproptosis and ceRNA were studied, which is expected to provide more effective and personalized treatment options for ESCA patients. The schematic diagram of the research design is shown in Figure 1.

## 2 METHOD

### 2.1 Data collection and preprocessing

The TCGA‐ESCA cohort data were acquired from the TCGA database (https://tcga‐data.nci.nih.gov/tcga/), with RNA‐seq and miRNA‐seq data utilized for gene expression evaluation. Clinical sample data were employed in subsequent receiver operating characteristic (ROC) analysis to assess the gene‐clinical data association.^32^ A total of five sequencing data sets containing ESCA samples were downloaded from the Gene Expression Omnibus (GEO) database (http://www.ncbi.nlm.nih.gov/geo/; GSE5364, GSE20347, GSE29001, GSE38129 and GSE63941) for subsequent gene expression and correlation analysis.^33^ In order to remove probes corresponding to multiple molecules from one probe, probes with the highest signal value were retained for those corresponding to the same molecule. Furthermore, differential analysis between the tumour group and the control group was carried out using the Limma package.^34^ The gene set related to m6A,^11^ glycolysis^35^, ^36^ and cuproptosis^28^ refers to previous studies.

### 2.2 Immunohistochemical (IHC) assay

This study presents a retrospective analysis of 26 patients diagnosed with ESCA who underwent surgical resection at Taihe Hospital between 2017 and 2019, with confirmation by pathological examination. Following standard protocols detailed in previous studies,^23^ IHC staining was utilized on both the tumour tissues and their adjacent counterparts, which were sectioned and processed for incubation with METTL3 antibody and corresponding secondary antibody. The IHC data were independently analysed by two professional pathologists utilizing a scoring system ranging from 0 (negative) to 3 (strongly positive). The Wilcoxon rank sum test was used to compare the expression differences of METTL3 between the tumour group and paracancerous group.

### 2.3 Functional enrichment analysis

Perform Pearson correlation analysis to investigate the association between the expression levels of METTL3 and other genes in the TCGA ESCA data set. To visually represent all correlation analysis outcomes, construct a volcanic map and show chord plots^37^ depicting the top 10 genes that exhibit a positive and negative correlation with METTL3, respectively. Furthermore, under the condition of cor >0.5 and *p* < 0.05, filter the co‐expressed genes of METTL3 based on retention of only the genes that encode proteins. Subsequently, we employed the clusterProfiler software package^38^ to conduct Gene Ontology (GO) and Kyoto Encyclopedia of Genes and Genomes (KEGG) enrichment analysis on the co‐expressed genes mentioned previously. We used ChiPlot to visualize the analysis results (http://www.chiplot.online/). Extract the expression data of METTL3 from the TCGA ESCA dataset, and categorize it into high and low expression groups according to its expression levels. Next, we utilized the DESeq2 package^39^ to undertake differential analysis on the original counts matrix of the TCGA ESCA data set. Employ the clusterProfiler package to execute gene set enrichment analysis (GSEA) on the differentially expressed genes mentioned above, with reference to the gene set: c2.cp.all.v2022.1.Hs.symbols.gmt [All Canonical Pathways] (3050). Finally, we used the ggplot2 package to visualize the outcomes of the aforementioned enrichment analysis. Data with the *p*.adj <0. 05 were considered statistically significant. The gene set database refers to MSigDB Collections (https://www.gsea‐msigdb.org/gsea/msigdb/collections.jsp).

### 2.4 Cell culture and treatment

The KYSE‐150 cell line, derived from human oesophageal carcinoma, was procured from the reputable BeNa Culture Collection (BNCC) under the identifier BNCC359343. Adhering to the recommended protocols, we successfully implemented the transfection of METTL3 siRNA into the malignant cells, employing the Lipofectamine 3000 transfection reagent (L3000015, Invitrogen). The specific siRNA sequences utilized in our experiments are elucidated in Table 1.

**TABLE 1:** The sequences of siRNAs used this study.

Gene	Sense	Antisense
METTL3 (human) siRNA‐772	AGAUGUUGAUCUGGAGAUATT	UAUCUCCAGAUCAACAUCUTT
METTL3 (human) siRNA‐1229	CAGUGGAUCUGUUGUGAUATT	UAUCACAACAGAUCCACUGTT
METTL3 (human) siRNA‐1012	CAGACGAAUUAUCAAUAAATT	UUUAUUGAUAAUUCGUCUGTT
METTL3 (human) siRNA‐857	GAGCUAUUAAAUACUACAATT	UUGUAGUAUUUAAUAGCUCTT
METTL3 (human) siRNA‐524	CAAGAUGAUGCACAUCCUATT	UAGGAUGUGCAUCAUCUUGTT
METTL3 (human) siRNA‐1609	GGAUUGUGAUGUGAUCGUATT	UACGAUCACAUCACAAUCCTT
NC	UUCUCCGAACGUGUCACGUTT	ACGUGACACGUUCGGAGAATT

### 2.5 Total RNA extraction and quantitative real‐time PCR

Total RNA was extracted from KYSE‐150 cell lines using Trizon Reagent (CW0580S, CWBIO). The cDNA was synthesized using HiScript II Q RT SuperMix for qPCR (R223‐01, Vazyme). Real‐time quantitative PCR (qRT‐PCR) was carried out using the CFX Connect™ system (Bio‐Rad) and the 2^−ΔΔCt^ method was employed to determine the relative expression levels. In order to normalize the data, β‐actin was utilized as the internal control. The detailed primer sequences can be found in Table S1.

### 2.6 EdU proliferation assay

EdU is a fluorescent marker for DNA synthesis, which can be selectively recognized and labelled by DNA synthase in cells. We used the EdU detection kit (C0078S, Beyotime) to stain according to the instructions provided by the manufacturer. Subsequently, observation and image analysis were conducted using a fluorescence microscope. For a detailed experimental protocol, please refer to our previous research.^40^

### 2.7 CCK‐8 assays

CCK‐8 reagent is a commonly used cell proliferation and cytotoxicity assessment reagent. We followed the instructions provided by the manufacturer for the CCK‐8 kit (KGA317, KeyGen) and co‐incubated it with cultured cells. CCK‐8 reagent can detect cellular metabolic activity through the activity of cell reductase. Finally, the absorbance value is measured using a spectrophotometer. The change in absorbance value reflects the activity of cell proliferation and can quantitatively evaluate the degree of cell proliferation.

### 2.8 Clone formation assay

Individual cells in the culture medium are homogenously dispersed in the culture dish, and over the course of culturing, they proliferate and form cell communities through cell division. After 1 week of cultivation, the cell communities grow into visible clones. As per the protocol, the cells are cleaned using PBS, fixed with paraformaldehyde and photographed. The proliferation rate of the cells can be assessed quantitatively by counting the number of clones formed.

### 2.9 Wound healing assay

We transfected cells with seed and culture them in a six‐well plate until they reach 90% confluence. Use a 200 μL pipette tip to create a scratch on the cells. Record and observe cell migration at 0, 24 and 48 h under a microscope. Utilize Image J software to quantify the area of the scratch.

### 2.10 Cellular flow cytometry analysis

Collect cultured cells and stain them using Cell Cycle Staining Kit (CCS012, MULTISCIENCES). Cell analysis was performed using flow cytometry to determine the proportion of G0/G1, S and G2/M phases of cells based on DNA content, in order to understand the distribution of cells at different cycle stages.

The Annexin V‐FITC/PI Cell Apoptosis Detection Kit (AP101, MULTI SCIENCES) is used to detect cell apoptosis. The proportion of apoptotic cells was calculated and statistically analysed using flow cytometry analysis and data processing software.

To evaluate the glucose uptake and metabolism of cells, we used the 2‐NBDG fluorescence labelled glucose experimental method. Cells were exposed to 2‐NBDG fluorescent probes in the culture medium. The 2‐NBDG fluorescence signals of cell uptake and metabolism can be detected and analysed by flow cytometry. By comparing with the control group, we were able to quantitatively evaluate the uptake and utilization of glucose by cells.

### 2.11 Measurement of lactate

The measurement of lactate levels in the culture medium was conducted using the Lactate Colorimetric Assay Kit (E‐BC‐K044‐M, Elabscience), with absorbance at 530 nm being measured in accordance with the manufacturer's instructions.

### 2.12 CeRNA network analysis

MiRNAs that are potentially capable of targeting METTL3 were predicted using TarBase (https://dianalab.e‐ce.uth.gr/html/diana/web/index.php?r=tarbasev8%2Findex)^41^ and mirDIP (http://ophid.utoronto.ca/mirDIP/index.jsp)^42^ platforms. Finalized target miRNA was determined through differential expression analysis. Similarly, ENCORI (https://rnasysu.com/encori/)^43^ and miRNet (https://www.mirnet.ca/)^44^ platforms were employed to predict lncRNAs capable of targeting the target miRNA, and the finalized target lncRNA was determined through differential expression analysis. Finally, a ceRNA network was constructed based on the ceRNA hypothesis. We utilized the RNAHybrid online tool to predict potential binding sites between mRNA‐miRNA and lncRNA‐miRNA.^45^

### 2.13 Statistics analysis

All statistical analyses in this study were performed using the Xiantao online database tool (https://www.xiantao.love) and R. The obtained data were analysed by one‐way or two‐way analysis of variance, while Kruskal–Wallis or Wilcoxon rank‐sum test was used for nonparametric data. *p* < 0.05 was considered statistically significant for all analyses.

## 3 RESULTS

### 3.1 Identification of dysregulated genes and diagnostic value of METTL3 in ESCA

**FIGURE 2:**
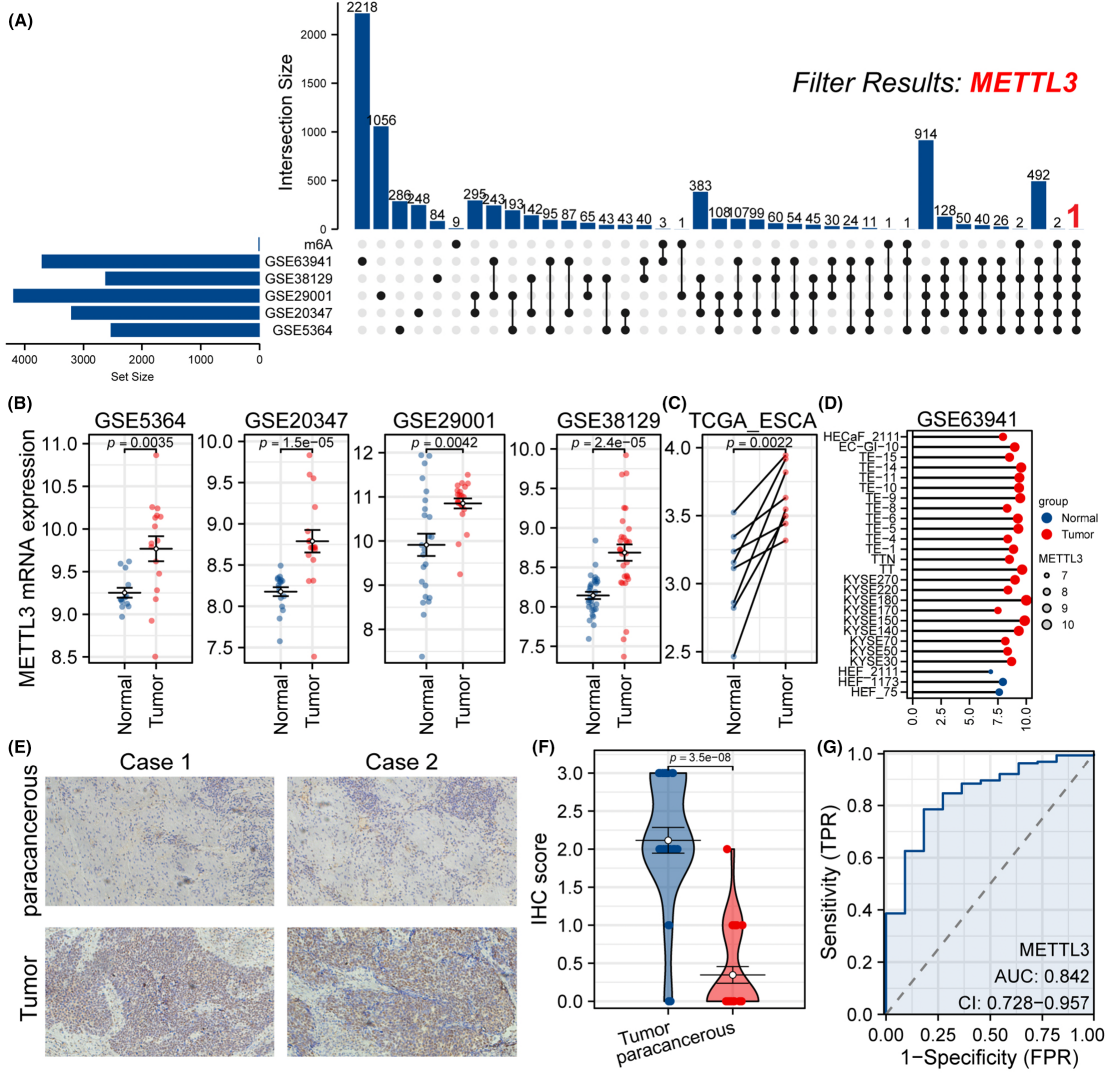
Identification of dysregulated genes and diagnostic value of METTL3 in ESCA. (A) The UpSet plot visualizes the target genes satisfying the screening criteria. (B) In all four GEO datasets, METTL3 expression was observed to be significantly higher in tumour samples than in the control group (*p* < 0.05). (C) Utilizing the TCGA paired sample data, it was found that the expression level of METTL3 in tumour tissues was remarkably higher than in the adjacent tissues (*p* < 0.05). (D) The expression of METTL3 was significantly elevated in ESCA cell lines compared to normal cells (*p* < 0.05). (E) The IHC assay was performed to evaluate METTL3 expression in tumour sections and paired paracancerous tissues obtained from ESCA patients. (F) Statistical analyses demonstrated a significant upregulation of METTL3 protein expression in ESCA patient samples relative to the paracancerous tissues (*p* < 0.05). (G) Diagnostic efficacy was assessed by ROC curves.

In order to identify dysregulated genes in ESCA, we performed differential analysis on five GEO datasets with filter criteria of |logFC| >0.5 and *p*.adj <0.05. The analysis of gene expression data sets revealed that there were 2529 differentially expressed genes in GSE5364, 3212 in GSE20347, 4194 in GSE29001, 2622 in GSE38129 and 3710 in GSE63941. Using UpSet plot, we discovered that only METTL3 intersected with the 20 m6A‐related genes across the five GEO data sets (Figure 2A). Group comparison plots demonstrated the expression difference of METTL3 between tumour and control groups in four GEO datasets (Figure 2B, *p* < 0.05). Moreover, The TCGA paired sample data analysis showed that the expression level of METTL3 was significantly higher in tumour tissues than in paired adjacent tissues (Figure 2C). Furthermore, increased levels of METTL3 were observed in ESCA cell lines, particularly in KYSE 180 and KYSE 150 (Figure 2D). IHC staining analysis indicated that METTL3 was significantly enriched in the nucleus, with statistical analysis confirming a significant increase of METTL3 protein expression in ESCA patient samples compared to paracancerous tissues (Figure 2E,F). To further investigate the diagnostic accuracy of METTL3 in ESCA, we constructed ROC curves and calculated the area under the curve (AUC). The ROC curve results demonstrated that METTL3 had a high diagnostic value for ESCA (AUC: 0.842; CI: 0.728−0.957; *p* < 0.05) (Figure 2G).

### 3.2 Correlation analysis and functional enrichment of co‐expressed genes with METTL3 in ESCA

**FIGURE 3:**
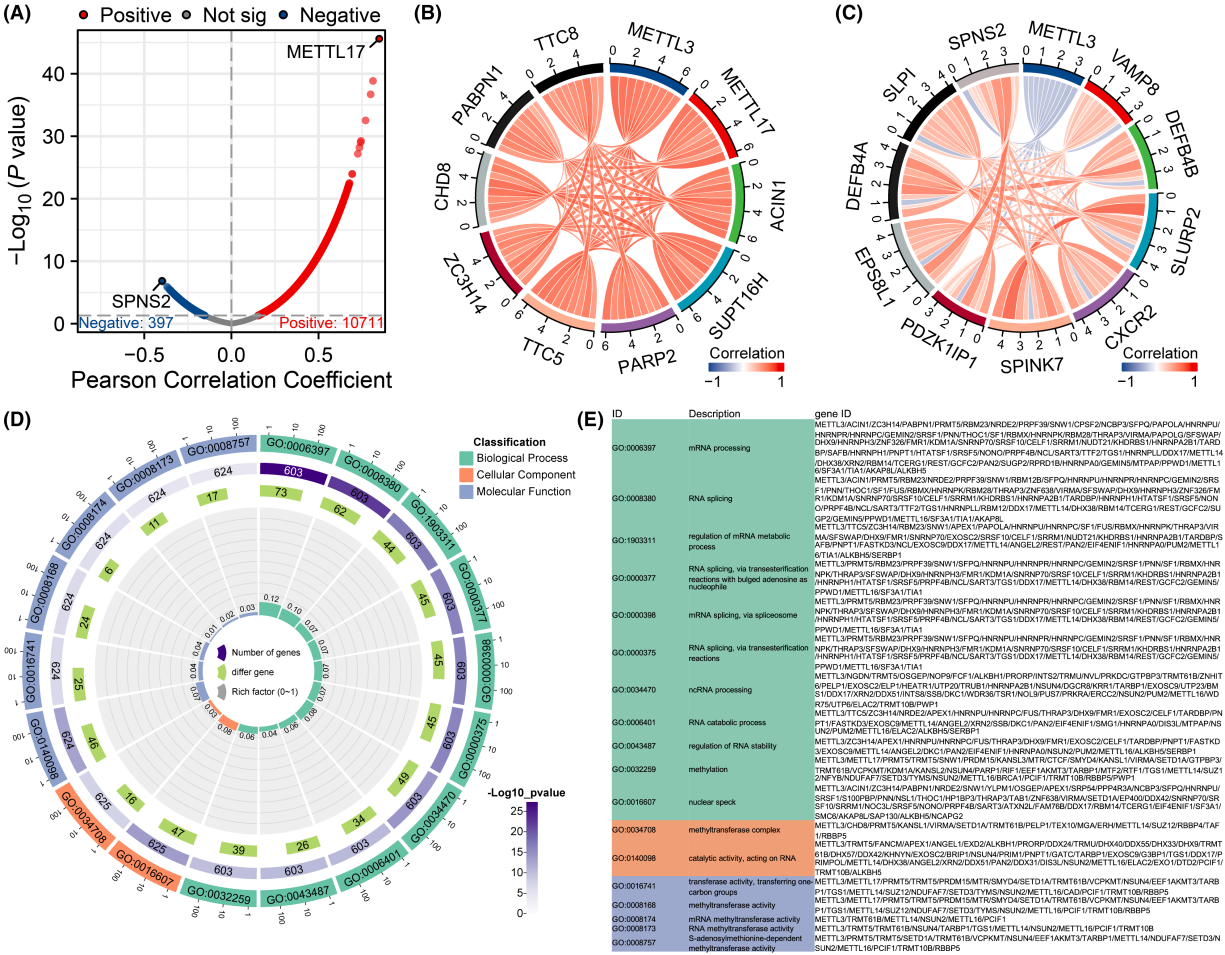
Correlation analysis and functional enrichment of co‐expressed genes with METTL3 in ESCA. (A) Map of volcanoes showing the correlation patterns of METTL3 with other genes. (B) Correlation analysis between METTL3 and the nine most positively correlated genes. (C) Correlation analysis between METTL3 and the nine most negatively correlated genes. (D) The circular diagram illustrates the potential biological pathways in which METTL3 and co‐expressed genes may be involved. (E) The comprehensive information of the biological pathways in which METTL3 and its co‐expressed genes may participate.

In order to gain a better understanding of the biological functions of METTL3, we utilized Pearson correlation analysis to examine the correlation between METTL3 and each gene in the TCGA ESCA data set. We found that, at a significance level of *p* < 0.05 and within the scope of protein‐coding genes, 10,711 genes exhibited a positive correlation with METTL3, while 397 genes displayed a negative correlation. Interestingly, the expression of METTL17 was found to have the highest positive correlation coefficient with METTL3, while SPNS2 exhibited the highest negative correlation coefficient (Figure 3A). Additionally, chord diagrams were constructed to identify the top 10 positively correlated genes (Figure 3B) and negatively correlated genes (Figure 3C) with METTL3. To narrow down the scope of our investigation, we set the threshold for correlation (cor) > 0.5 and *p* < 0.05, ultimately identifying a total of 657 co‐expressed genes. We conducted further GO and KEGG enrichment analysis on these co‐expressed genes, revealing that there are 58 biological pathways that include the METTL3 gene (50 biological process, two cellular component and six molecular function, *p*.adj <0.05). Our results suggest that METTL3 and co‐expressed genes may be involved in mRNA processing, nuclear speck and catalytic activity, acting on RNA (Figure 3D,E).

### 3.3 GSEA analysis reveals METTL3‐related pathways

**FIGURE 4:**
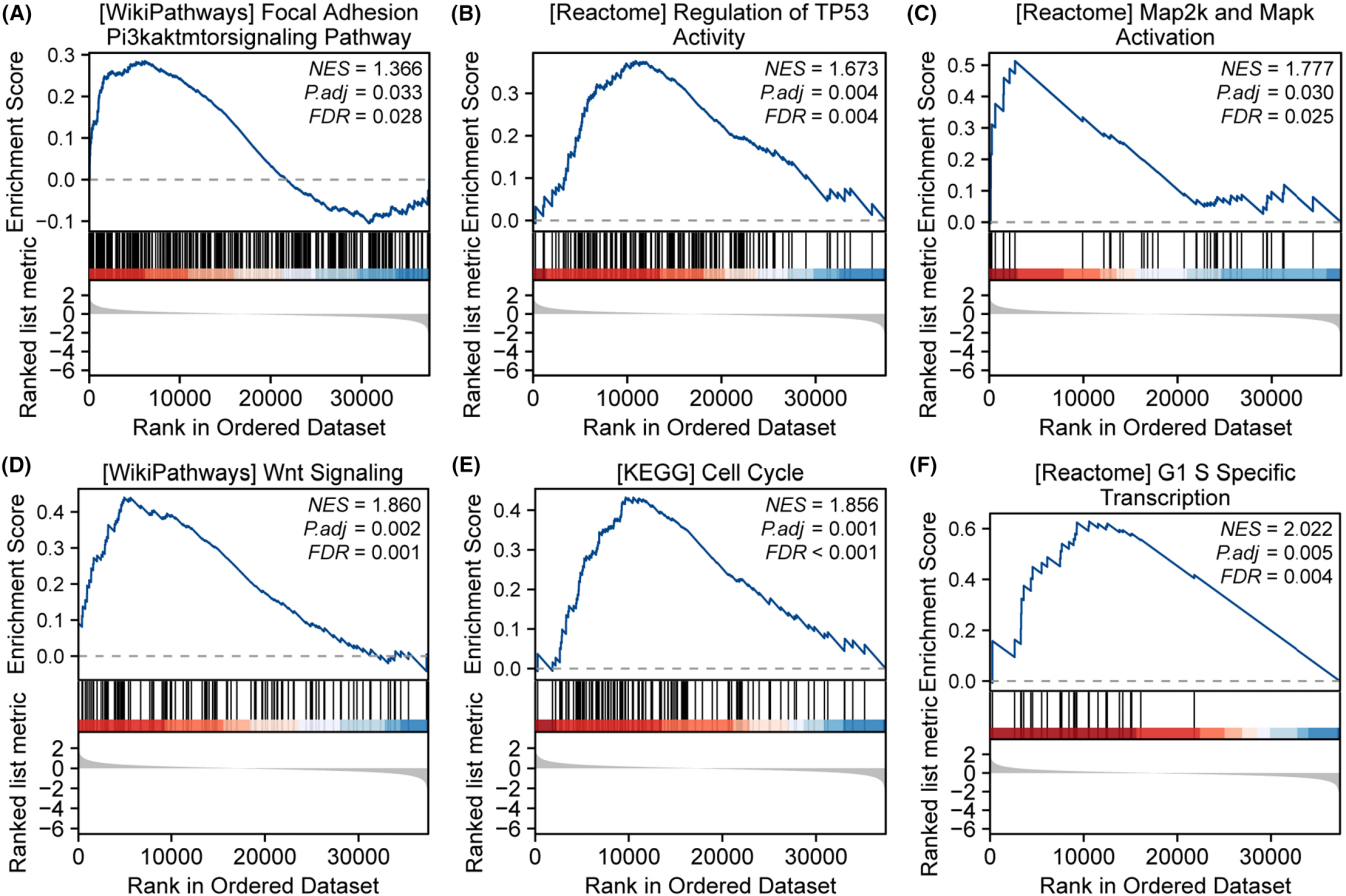
GSEA analysis reveals METTL3‐related pathways. (A) PI3K/AKT/MTOR signalling pathway, (B) regulation of TP53 activity, (C) MAP2K and MAPK activation, (D) WNT signalling, (E) cell cycle and (F) G1/S specific transcription.

To further explore the potential functions of METTL3, we conducted GSEA. ESCA cohort samples were divided into high and low expression groups based on the expression levels of METTL3 to identify differential expression gene sets related to METTL3. The GSEA results showed that a total of 297 gene sets were filtered out under the conditions of |NES| > 1 and *p*.adj <0.05. Among them, we found that PI3K/AKT/MTOR signalling pathway (NES = 1.37, *p*.adj <0.05), regulation of TP53 Activity (NES = 1.67, *p*.adj <0.05), MAP2K and MAPK activation (NES = 1.78, *p*.adj <0.05), WNT signalling (NES = 1.86, *p*.adj <0.05), cell cycle (NES = 1.86, *p*.adj <0.05) and G1/S specific transcription (NES = 2.02, *p*.adj <0.05) were upregulated in the METTL3 high expression cluster (Figure 4).

### 3.4 Inhibition of METTL3 suppresses ESCA cell proliferation

**FIGURE 5:**
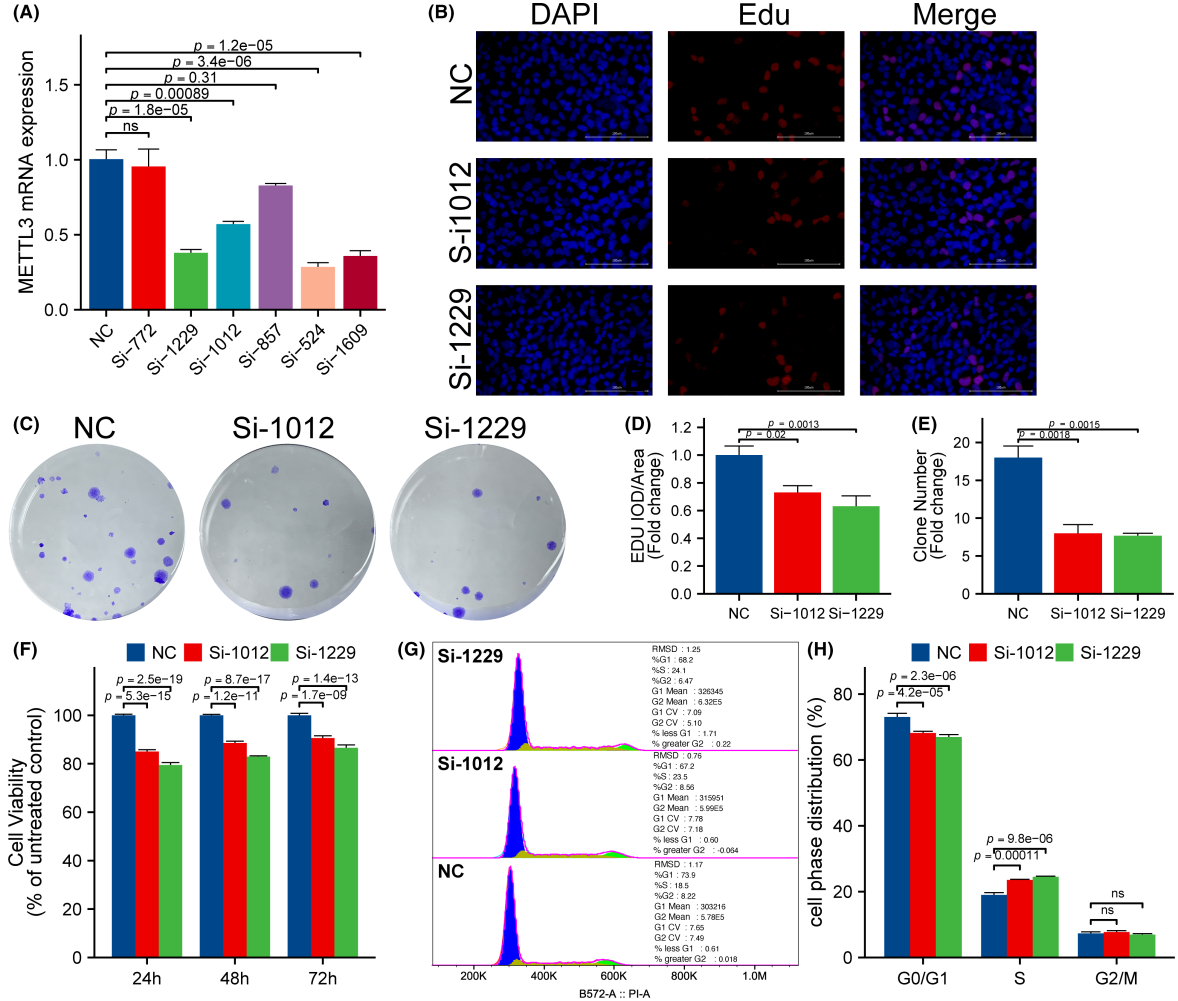
Inhibition of METTL3 Suppresses ESCA Cell Proliferation. (A) The interference efficiency of six siRNAs was validated through qRT‐PCR experiments. The results obtained from EdU (B, D), clone formation (C, E) and CCK‐8 assays (F) demonstrated that the experimental group displayed a significant reduction in cellular viability compared to the control group. (G, H) Flow cytometry analysis revealed that compared with the control group, the tumour cells in the experimental group were blocked in S phase.

To investigate the inhibitory effect of disrupting METTL3 expression on ESCA cells, we designed six different siRNA sequences to interfere with METTL3 expression. qRT‐PCR experiments confirmed the interference efficiency of these six siRNAs (Figure 5A). Finally, SiRNA‐1012 (Si‐1012) and SiRNA‐1229 (Si‐1229) were selected as the experimental group for subsequent experiments. EdU proliferation assay, CCK‐8 assay and clone formation assay were used to measure cell proliferation activity. In the EdU assay, the experimental group showed a significant decrease in cell vitality compared with the control group (Figure 5B,D, *p* < 0.05). In the clone formation assay, cell activity in the experimental group was significantly reduced compared with the control group (Figure 5C,E, *p* < 0.05). In the CCK‐8 assay, the cell vitality of the experimental group was significantly lower than that of the control group (Figure 5F, *p* < 0.05). Interestingly, flow cytometry analysis revealed that compared with the control group, the tumour cells in the experimental group were blocked in S phase (Figure 5G,H, *p* < 0.05), corresponding to the results in Figure 4F, indicating that inhibiting METTL3 expression may interfere with the normal cell cycle progression of tumour cells.

### 3.5 METTL3 expression affects cell apoptosis and wound healing

**FIGURE 6:**
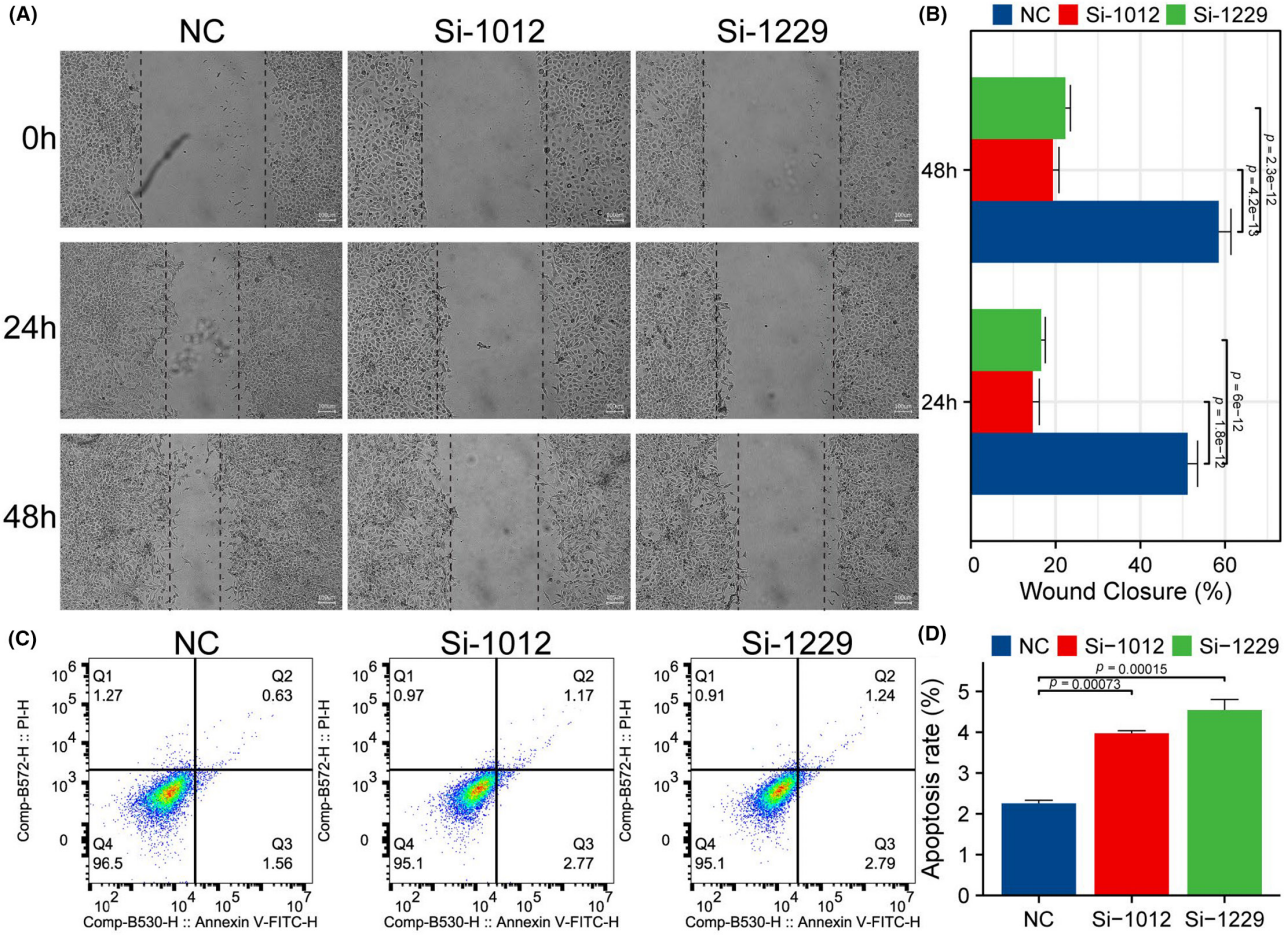
METTL3 expression affects cell apoptosis and wound healing. (A, B) The results showed that interference of METTL3 expression significantly inhibited wound healing rate in the experimental group. (C, D) The results showed that interference of METTL3 expression significantly increased the number of apoptotic cells in the experimental group.

This study investigated the role of METTL3 in wound healing and cell apoptosis through wound healing assay and flow cytometry. The results showed that interference of METTL3 expression significantly inhibited wound healing rate (Figure 6A,B, *p* < 0.05) and increased the number of apoptotic cells (Figure 6C,D, *p* < 0.05) in the experimental group. This suggests that METTL3 may be involved in biological processes such as immune inflammatory response, tissue regeneration and cell apoptosis.

### 3.6 METTL3 expression affects glycolysis in tumours

**FIGURE 7:**
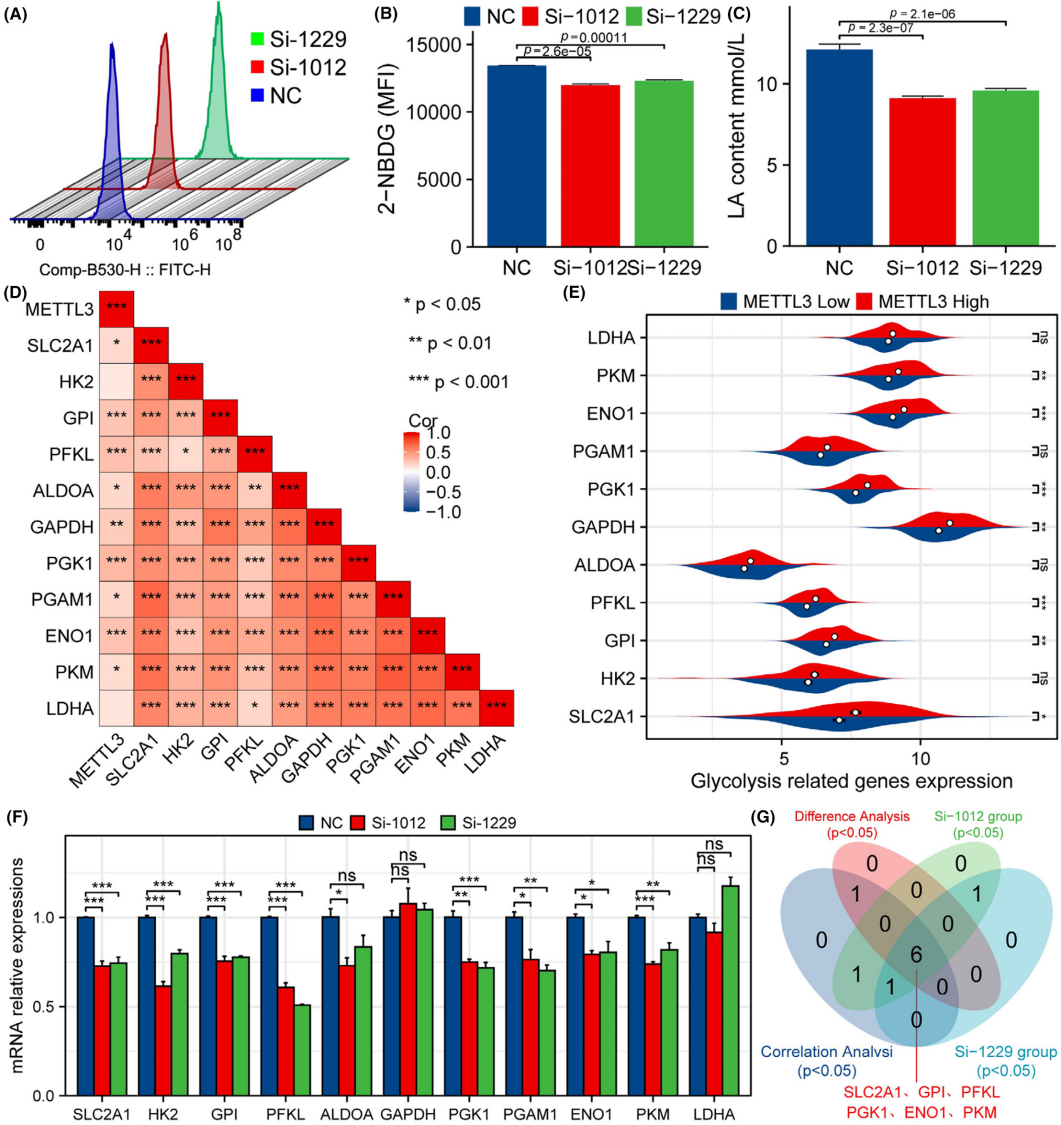
METTL3 expression affects glycolysis in tumours. (A, B) Interference with METTL3 expression can significantly reduce the uptake of 2‐NBDG in ESCA cells. (C) Interference with METTL3 expression can significantly reduce the lactate production in ESCA cells. (D) The TCGA ESCA data set analysed the correlation of METTL3 with 11 glycolysis‐related genes. (E) The expression differences of 11 glycolysis‐related genes between high and low expression groups of METTL3. (F) The expression differences of 11 glycolysis‐related genes between the experimental group and the control group in the siRNA transfection experiment. (G) The Venn diagram further revealed positive genes matching with the four studies mentioned above, namely SLC2A1, GPI, PFKL, PGK1, ENO1 and PKM.

In previous studies, we found a significant positive correlation between METTL3 expression and ^18^F‐FDG PET/CT metabolic parameters. Therefore, the present study aimed to further explore the relationship between METTL3 expression and tumour cell glycolysis. Experimental investigations revealed that interfering with METTL3 expression significantly reduced the uptake of 2‐NBDG (Figure 7A,B, *p* < 0.05) and lactate production (Figure 7C, *p* < 0.05) in ESCA cells. Analysis of TCGA ESCA data set results showed a strong positive correlation between ESCA expression and several glycolysis‐related genes such as SLC2A1, GPI, PFKL, ALDOA, GAPDH, PGK1, PGAM1, ENO1, PKM and LDHA (Figure 7D, *p* < 0.05). Additionally, in the high expression group of METTL3, the expression levels of SLC2A1, GPI, PFKL, GAPDH, PGK1, ENO1 and PKM were significantly higher than those in the low expression group (Figure 7E, *p* < 0.05). Furthermore, siRNA transfection experiments showed a significant downregulation of SLC2A1, HK2, GPI, PFKL, PGAM1 and ENO1 gene expression in the experimental group as compared to the control group, while PKM and LDHA gene expression only exhibited a significant decrease in the Si‐1012 group (Figure 7F, *p* < 0.05). The Venn diagram further revealed positive genes matching with the four studies mentioned above, namely SLC2A1, GPI, PFKL, PGK1, ENO1 and PKM (Figure 7G). These results suggest that METTL3 may regulate tumour cell glycolysis by modulating the expression of glycolysis‐related genes. Such findings provide new insights and directions for future research in the field of tumour metabolism.

### 3.7 METTL3 expression correlated with cuproptosis‐related genes in ESCA

**FIGURE 8:**
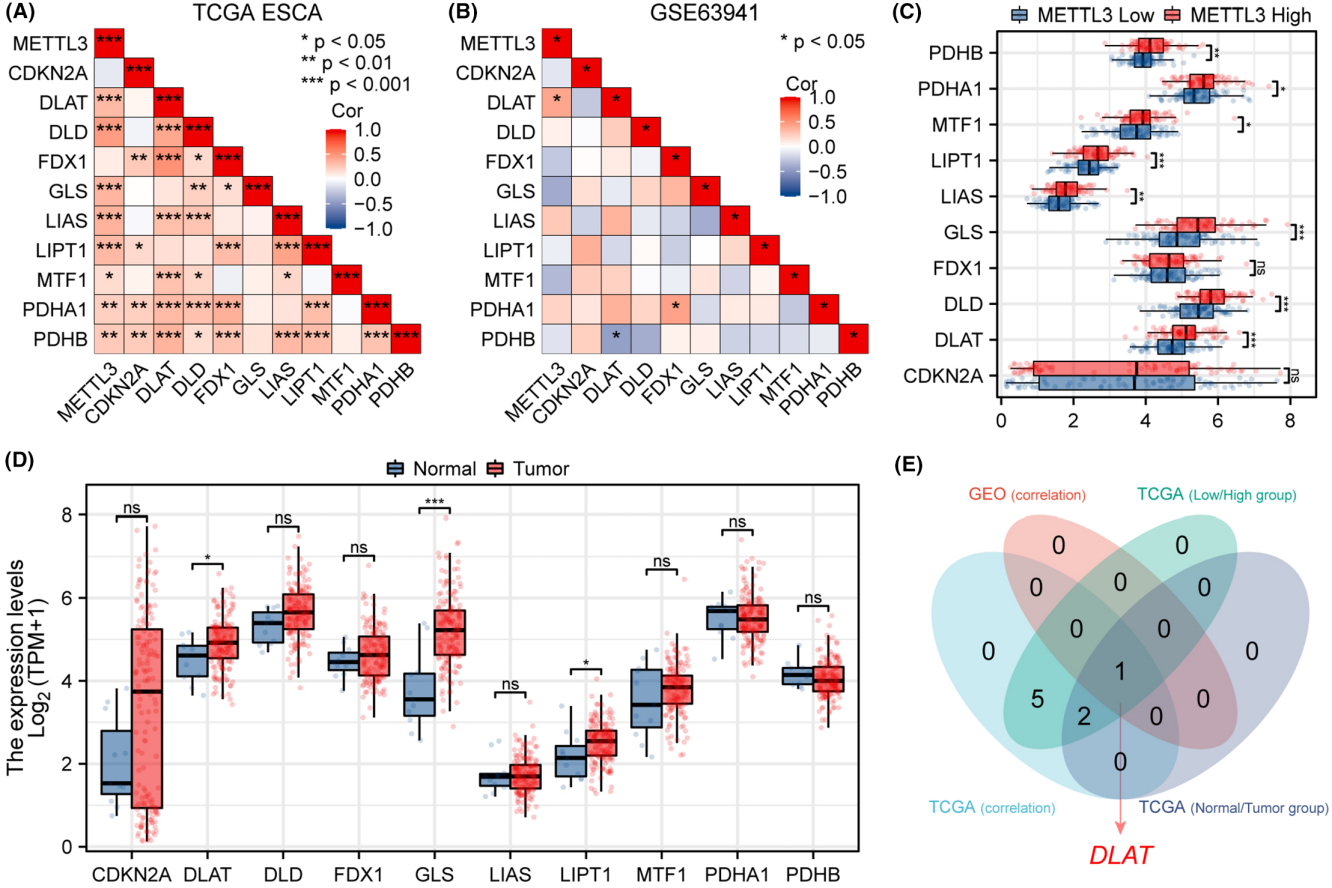
METTL3 expression correlated with cuproptosis‐related genes in ESCA. (A) The expression correlation between METTL3 and 10 cuproptosis‐related genes in the TCGA ESCA data set. (B) The expression correlation between METTL3 and 10 cuproptosis‐related genes in the GSE63941 data set. (C) The expression differences of 10 cuproptosis‐related genes between high and low expression groups of METTL3. (D) The expression differences of 10 cuproptosis‐related genes between tumour samples and normal samples in the TCGA ESCA data set. (E) The Venn diagram further revealed positive genes matching with the four studies mentioned above, namely DLAT.

Cuproptosis is a process of programmed cell death induced by copper ions, which plays a vital role in tumorigenesis and progression. This study analysed the TCGA ESCA cohort and GSE63941 dataset to investigate the correlation between METTL3 expression and cuproptosis‐related gene expression. The results showed a significant positive correlation between METTL3 expression and DLAT in both TCGA ESCA cohort and GSE63941 data set (Figure 8A,B, *p* < 0.05). Moreover, in the TCGA ESCA cohort data, METTL3 expression was positively correlated with DLD, GLS, LIAS, LIPT1, MTF1, PDHA1 and PDHB (Figure 8A, *p* < 0.05). To determine whether cuproptosis is associated with changes in METTL3 expression, we further studied the expression of cuproptosis‐related genes between METTL3 high and low expression groups. The results showed that the expression of DLAT, DLD, GLS, LIAS, LIPT1, MTF1, PDHA1 and PDHB was increased in the high expression group (Figure 8C, *p* < 0.05). Additionally, we also investigated the expression of cuproptosis‐related genes between tumour and normal samples in the TCGA ESCA cohort and found that the expression of DLAT, GLS and LIPT1 was increased in tumour samples (Figure 8D, *p* < 0.05). Finally, the Venn diagram showed that only DLAT met all the aforementioned screening criteria (Figure 8E). In conclusion, this study found a potential correlation between METTL3 and cuproptosis in tumorigenesis and progression, which may provide new insights and targets for cancer prevention and treatment.

### 3.8 METTL3 and hsa‐miR‐101‐3p interaction in ESCA

**FIGURE 9:**
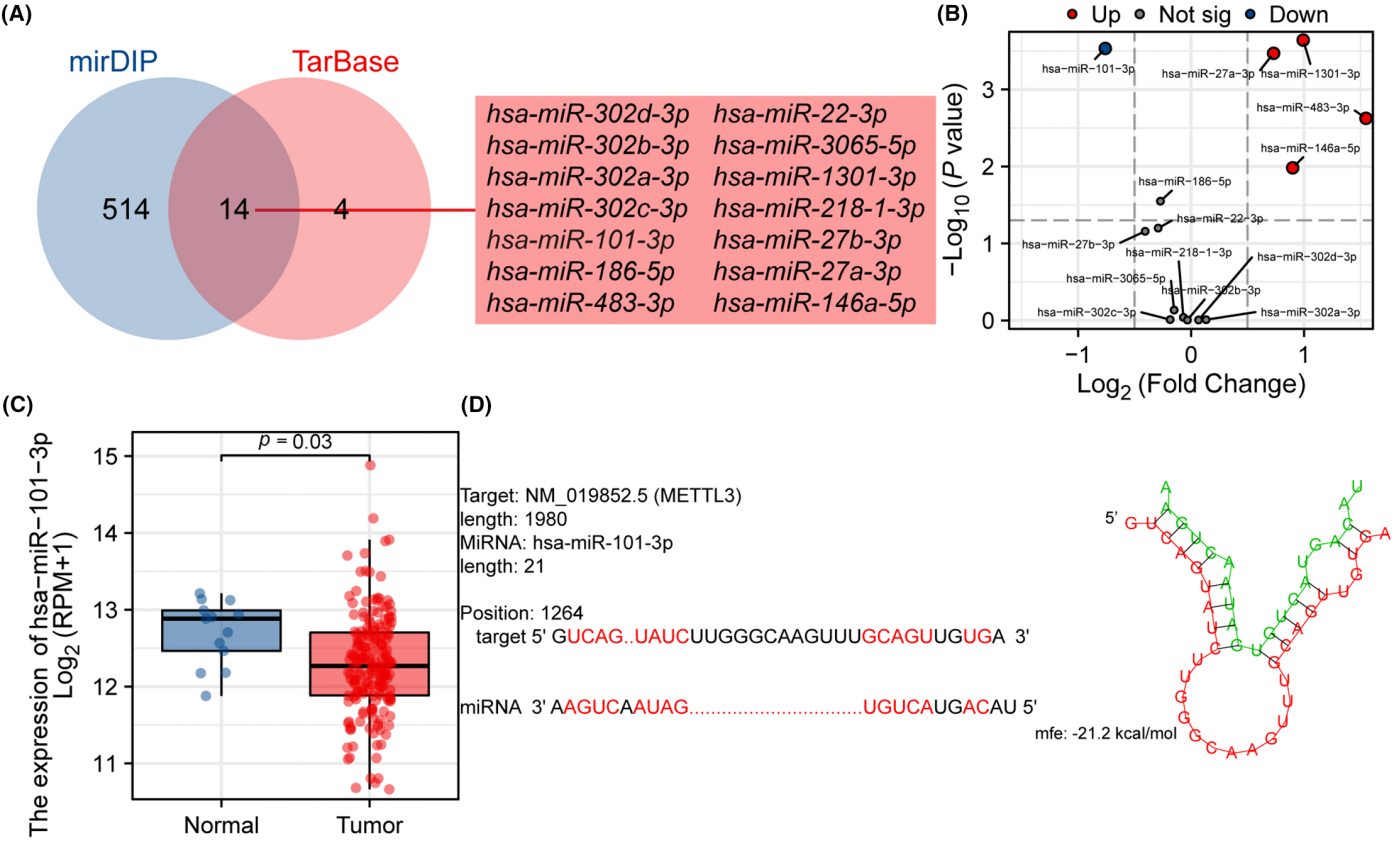
METTL3 and hsa‐miR‐101‐3p interaction in ESCA. (A) Venn diagram demonstrated that 14 miRNAs co‐exist in both databases. (B) Differential expression of interacted miRNA in TCGA ESCA cohort. (C) The expression of hsa‐miR‐101‐3p in tumour samples from the TCGA ESCA data set was significantly lower than that in normal samples. (D) The RNAHybrid online tool predicts potential binding sites between METTL3 and hsa‐miR‐101‐3p.

Previous studies have indicated that the ceRNA mechanism plays a significant role in the occurrence and development of oesophageal cancer. In this study, we analysed the METTL3‐related ceRNA network in ESCA. Using the mirDIP and TarBase databases, we predicted 528 and 18 miRNAs, respectively, that interact with METTL3. The Venn diagram demonstrated that 14 miRNAs co‐exist in both databases (Figure 9A). Figure 9B displays the expression of five differentially expressed miRNAs, including hsa‐miR‐101‐3p, hsa‐miR‐1301‐3p, hsa‐miR‐27a‐3p, hsa‐miR‐483‐3p and hsa‐miR‐146a‐5p. According to the ceRNA hypothesis principle, the expression of miRNA and mRNA is inversely correlated. We selected hsa‐miR‐101‐3p as the targeted miRNA of METTL3. Figure 9C shows that the expression of hsa‐miR‐101‐3p in tumour samples is significantly lower compared to normal samples. Using RNAHybrid for bioinformatic analysis, potential binding sites between METTL3 and hsa‐miR‐101‐3p were identified (Figure 9D).

### 3.9 Identification of lncRNAs interacting with hsa‐miR‐101‐3p in ESCA

**FIGURE 10:**
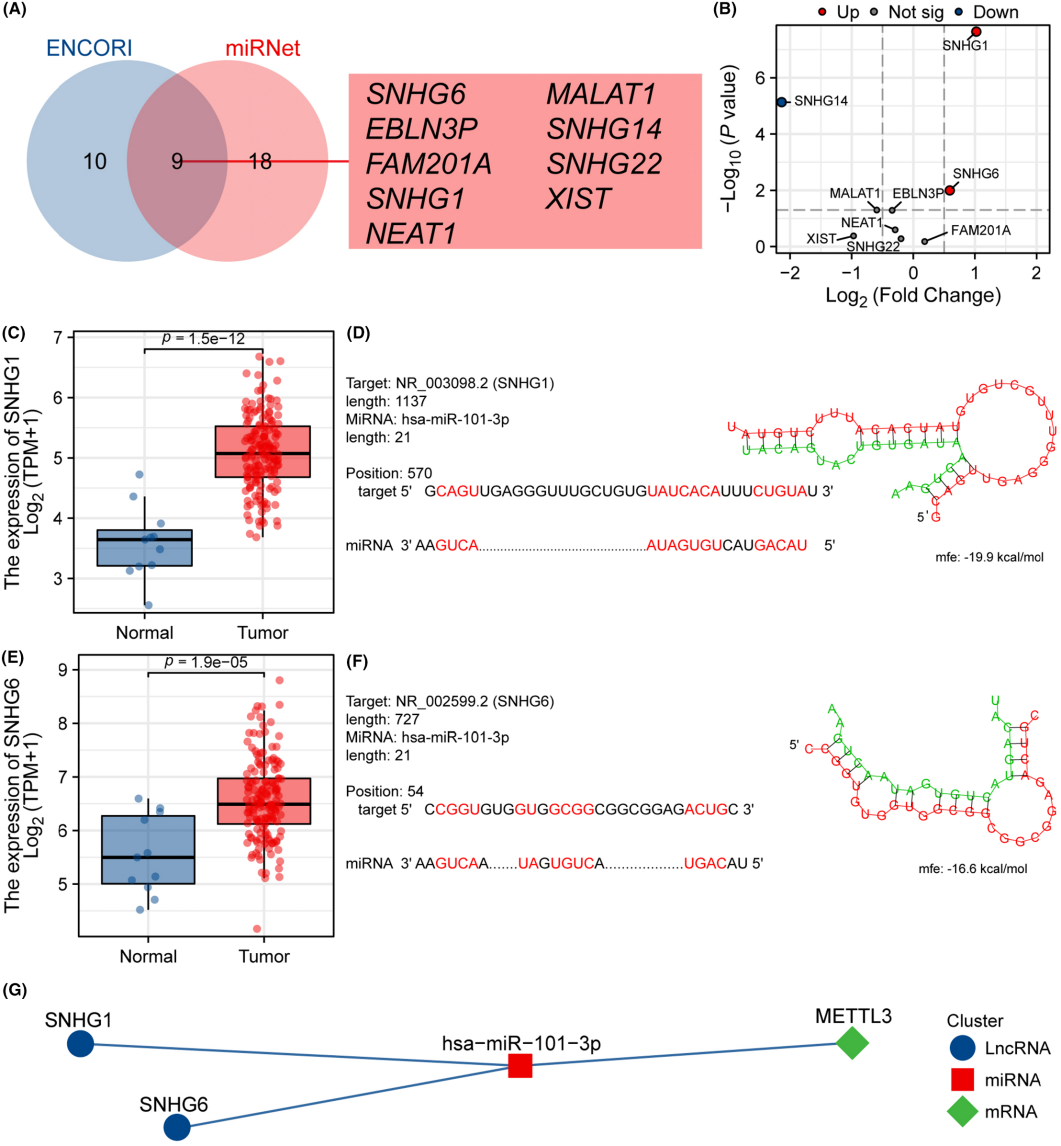
Identification of lncRNAs interacting with hsa‐miR‐101‐3p in ESCA. (A) Venn diagram demonstrated that nine lncRNAs co‐exist in both databases. (B) Differential expression of interacted lncRNA in TCGA ESCA cohort. (C, E) The expression of SNHG1 and SNHG6 in tumour samples from the TCGA ESCA data set was significantly lower than that in normal samples. (D, F) The RNAHybrid online tool predicts the potential binding sites of hsa‐miR‐101‐3p with SNHG1 and SNHG6, respectively. (G) The network diagram shows the relationship of the final ceRNA network.

After analysing the ENCORI and miRNet databases, we predicted the existence of 19 and 27 lncRNAs that interact with hsa‐miR‐101‐3p, respectively. Among them, nine lncRNAs were found to appear in both databases (Figure 10A). Figure 10B illustrates the differential expression of three lncRNAs, namely SNHG14, SNHG1 and SNHG6. Based on the ceRNA hypothesis, the expression of miRNA and lncRNA is negatively correlated, and thus, we selected SNHG1 and SNHG6 as the target lncRNAs of hsa‐miR‐101‐3p. Figure 10C,E, respectively, show the significantly higher expression levels of SNHG1 and SNHG6 in tumour samples than in normal samples. Biological informatics analysis using RNAHybrid revealed that SNHG1 and SNHG6 have potential binding sites with hsa‐miR‐101‐3p (Figure 10D,F). These data indicate that SNHG1 and SNHG6 may act as ceRNAs to competitively bind hsa‐miR‐101‐3p and promote the expression of METTL3 (Figure 10G). Our study provides new insights into the role of METTL3 and its ceRNA network in ESCA.

## 4 DISCUSSION

**FIGURE 11:**
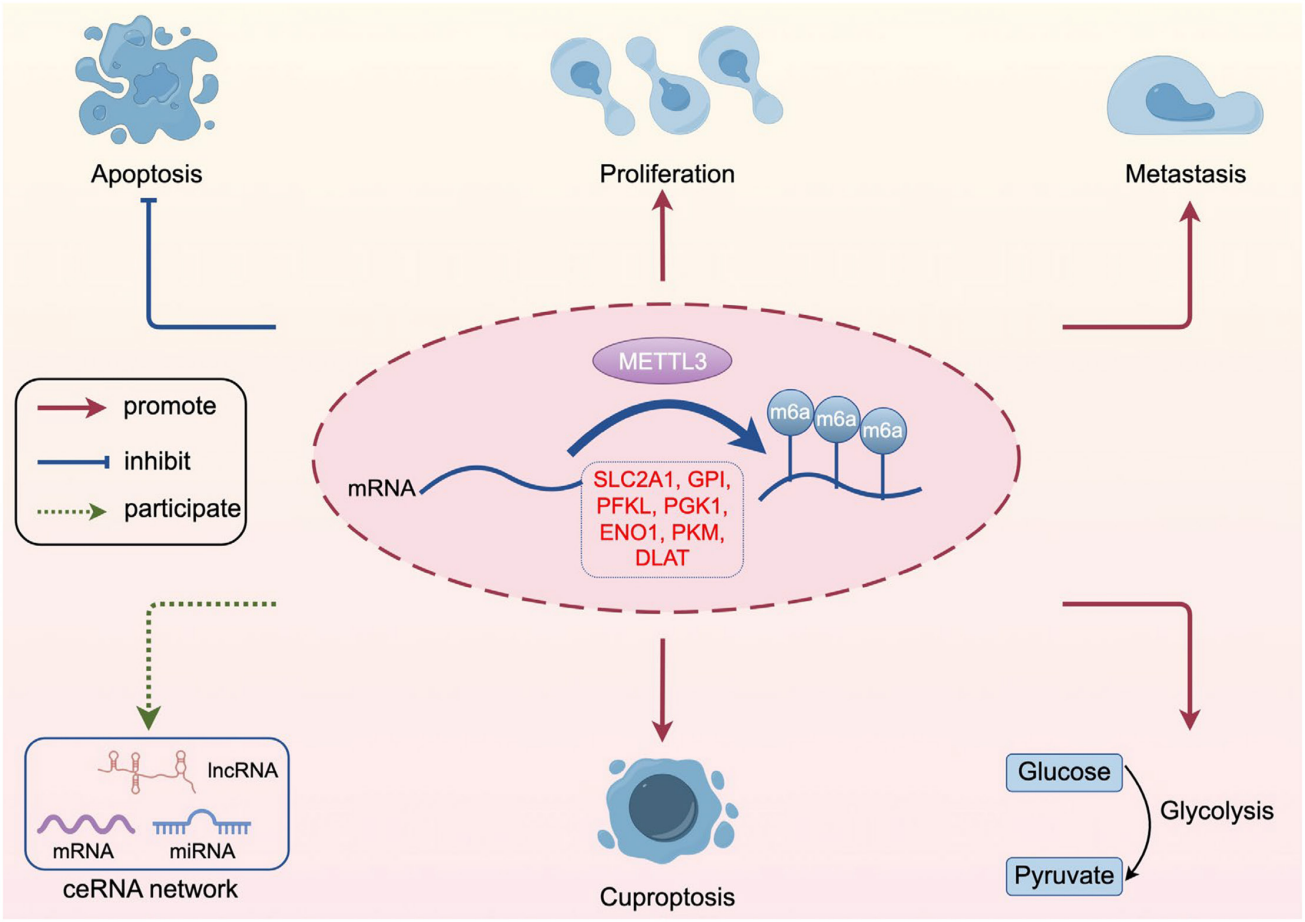
Research mechanism diagram. The figure was created by Figdraw (http://www.figdraw.com).

The high mortality and poor prognosis of ESCA highlight the importance of identifying reliable biomarkers for early detection and effective treatment.^2^, ^3^, ^4^ METTL3, an important RNA methyltransferase, plays a critical role in RNA modification, and its expression and catalysed RNA m6A modification are associated with the occurrence and progression of various human diseases.^10^, ^11^, ^12^, ^14^ Prior studies have demonstrated that METTL3 is overexpressed in various cancers and contributes to cancer progression and metastasis.^21^, ^46^, ^47^, ^48^, ^49^, ^50^, ^51^, ^52^, ^53^, ^54^, ^55^ In this study, we further confirmed through multi‐data set analysis that the expression of METTL3 was significantly higher in tumour samples than in the control group. ROC results indicated that its expression level has high accuracy in diagnosing ESCA. These findings further support the potential involvement of overexpressed METTL3 in ESCA cell development and progression. The research mechanism is depicted in Figure 11.

In the analysis of METTL3 co‐expression in the TCGA ESCA dataset, we found that METTL17 expression was positively correlated with METTL3 expression. Studies have shown that METTL17 is localized to mitochondria and has specific interactions with mitochondrial ribosomal RNA and the small subunit of mitochondrial ribosomes. Deletion of METTL17 leads to a decrease in m4C840 and m5C842 of 12S mitochondrial ribosomal RNA, affecting translation of mitochondrial protein‐coding genes and changes in oxidative phosphorylation metabolism products, ultimately leading to a decrease in embryonic stem cell pluripotency.^56^ METTL17 can act as a coactivator of ERs, and inhibition of METTL17 leads to reduced transcriptional activity of ERα and ERβ in breast cancer cells, reduces the expression of ER target genes and limits the growth of breast cancer cells.^57^ These results suggest that METTL17 may have similar biological functions as METTL3. Additionally, we found that SPNS2 expression was negatively correlated with METTL3 expression. SPNS2 is a Sphingosine‐1‐phosphate (S1P) transporter that activates lipid signalling cascades by exporting S1P. Regulating SPNS2 activity may help to treat cancer, inflammation and immune diseases.^58^ Studies have shown that the role of SPNS2 in tumours is dual‐sided, with increased expression reported in liver^59^, ^60^ and colorectal^61^, ^62^ cancer, where overexpression of SPNS2 promotes tumour cell development. Interestingly, studies have also found that upregulation of SPNS2 expression can inhibit the development of triple‐negative breast cancer cell lines.^63^ Bradley et al.^64^ reported that overexpression of SPNS2 can lead to apoptosis of non‐small cell lung cancer cells, while knocking out SPNS2 increases tumour cell mobility. Downregulation of SPNS2 is a potential risk factor for non‐small cell lung cancer. However, there are no reports on the relationship between METTL17 and SPNS2 genes with ESCA. We then performed GO and KEGG enrichment analyses on the co‐expression gene set, and several significant categories were enriched in the positively correlated group, including mRNA processing, nuclear speck and catalytic activity acting on RNA. Furthermore, GSEA suggests that METTL3 may be involved in various signalling pathways, including PI3K/AKT/MTOR signalling, regulation of TP53 activity, MAP2K and MAPK activation, WNT signalling, cell cycle and G1/S‐specific transcription. These results suggest that METTL3 may play an important biological role in tumorigenesis and development. In this article, we focus on the relationship between METTL3 and tumour cell proliferation, migration, glycolysis and cuproptosis.

Our experiment revealed that inhibiting the expression of METTL3 significantly arrested cells in the S phase of the cell cycle. Results from EdU, CCK‐8, cell cloning, scratch and apoptosis assays indicated that downregulation of METTL3 expression significantly inhibited proliferation and migration while promoting apoptosis in ESCA cells, consistent with previous findings.^65^, ^66^, ^67^ Our earlier study observed a significant positive correlation between the expression levels of METTL3 and 18F‐FDG metabolic parameters, suggesting a potential link between the expression of METTL3 and glycolytic ability in ESCA cells.^23^ In this study, we employed 2‐NBDG uptake and lactate production assays to demonstrate that inhibition of METTL3 expression significantly suppressed glycolysis in ESCA cells. Compared with previous studies,^68^ we conducted comprehensive bioinformatic analysis to investigate the potential relationship between METTL3 and 11 glycolysis‐related genes. Our qRT‐PCR experiments further revealed that METTL3 may promote the glycolysis capacity of ESCA cells by upregulating the expression of glycolysis‐related genes SLC2A1, GPI, PFKL, PGK1, ENO1 and PKM, thus facilitating tumour cell proliferation and migration. However, our study has several limitations. For instance, we primarily focused on in vitro experiments and additional in vivo studies are required to confirm our findings. Moreover, further research is needed to elucidate the regulatory mechanism of glycolysis mediated by METTL3 in ESCA cells. Overall, our results provide a basis for future efforts to develop novel therapeutic strategies for ESCA.

Cuproptosis is a copper‐dependent form of cell death that is implicated in the pathogenesis of various diseases, including cancer.^29^, ^69^, ^70^, ^71^ However, there have been no studies investigating the relationship between METTL3 and cuproptosis. In this study, we conducted a comprehensive bioinformatics analysis to explore the potential links between METTL3 and 10 cuproptosis‐related genes. Through our analysis of TCGA ESCA and GSE63941 data sets, we found a correlation only between expression levels of DLAT and METTL3. Furthermore, we observed that DLAT expression was significantly higher in tumour samples compared to normal samples, consistent with previous reports indicating DLAT upregulation in various types of cancer such as LIHC, LUAD, LUSC, PAAD and STAD.^72^, ^73^, ^74^, ^75^ Additionally, we found that DLAT expression was significantly higher in the high METTL3 expression group than in the low expression group, highlighting the potential association between these two genes in cuproptosis‐related tumour development. DLAT is a protein that exists in the inner membrane of mitochondria and serves as the E2 component of PDC. It plays a crucial role in the conversion of pyruvate to acetyl‐CoA in the mitochondrial matrix.^76^ DLAT is considered a potential target for cancer therapy. For example, Chen et al.^77^ demonstrated that PM2.5‐induced DLAT overexpression and enhanced glycolysis promoted non‐small cell lung cancer tumour formation. Goh et al.^78^ found that interfering with DLAT expression in gastric cancer cells using siRNA reduced tumour cell proliferation. Therefore, the correlation between DLAT and METTL3 in cuproptosis‐related tumour development may provide a new strategy for cancer prevention and treatment. In summary, our study is the first to propose a potential link between METTL3 and DLAT in cuproptosis‐related tumour development. However, further research is needed to elucidate the underlying mechanisms and verify the therapeutic potential of targeting this pathway in cancer.

CeRNA mechanism refers to a regulatory mechanism wherein RNA molecules with similar miRNA response elements compete to bind miRNAs.^30^ It plays a critical role in the development and progression of tumours.^31^ Some studies have shown that the ceRNA mechanism can affect processes such as tumour growth, malignancy, metastasis and recurrence by modulating the binding of miRNA to target RNAs.^31^, ^79^ Previous studies have demonstrated that METTL3 has an important role in the ceRNA network of breast cancer.^22^ However, no studies to date have investigated the relationship between METTL3 and ceRNA in ESCA. In this study, we first screened for miRNAs that interacted with METTL3 using different databases and performed differential analysis and target prediction to identify has‐miR‐101‐3p. Then, we predicted lncRNAs that could target the miRNA, selected and confirmed SNHG1 and SNHG6 as the lncRNAs targeting has‐miR‐101‐3p based on the ceRNA theory, and ultimately constructed two ceRNA networks, SNHG1/has‐miR‐101‐3p/METTL3 and SNHG6/has‐miR‐101‐3p/METTL3. However, while previous studies have confirmed the relationship between SNHG1 and SNHG6 and has‐miR‐101‐3p,^80^, ^81^, ^82^, ^83^, ^84^, ^85^, ^86^ this study is the first to predict and propose a miRNA targeting METTL3 in ESCA and reveal the lncRNAs that regulate miRNA expression and affect METTL3 expression, providing potential therapeutic targets for molecular treatment of ESCA.

## 5 CONCLUSION

In summary, this study has identified RNA methyltransferase METTL3 as a potential biomarker for ESCA detection, and its overexpression is associated with tumour development and progression. Our findings suggest that METTL3 may play a key role in tumorigenesis and progression by promoting tumour cell proliferation, migration, glycolysis and cuproptosis. Additionally, we identified a potential link between METTL3 and DLAT in cuproptosis‐related tumour development, providing a new strategy for cancer prevention and treatment. Furthermore, we constructed ceRNA networks involving METTL3, miRNA and lncRNAs, highlighting potential therapeutic targets for molecular treatment of ESCA. However, further research is needed to elucidate the underlying mechanisms and verify the therapeutic potential of targeting these pathways in ESCA. Overall, our study provides new insights into the pathogenesis of ESCA and offers potential avenues for the development of novel therapeutic strategies.

## AUTHOR CONTRIBUTIONS

**Xu‐Sheng Liu:** Conceptualization (equal); methodology (equal); writing – original draft (lead). **Yu Zhang:** Conceptualization (equal); methodology (equal). **Zi‐Yue Liu:** Conceptualization (equal); methodology (equal). **Yan Gao:** Software (equal); validation (equal). **Ling‐ling Yuan:** Conceptualization (equal); validation (equal). **Dao‐Bing Zeng:** Formal analysis (equal); investigation (equal). **Fan Tan:** Formal analysis (equal); investigation (equal). **Hua‐Bing Wan:** Formal analysis (equal); investigation (equal). **Zhi‐Jun Pei:** Supervision (lead); writing – review and editing (lead).

## CONFLICT OF INTEREST STATEMENT

All authors declare that they have no known competing financial interests or personal relationships that could have appeared to influence the work reported in this paper.

## Supporting information


Table S1.

## Data Availability

The data sets generated during and/or analysed during the current study are available from the corresponding author on reasonable request.
